# Gender differences in the ideal cutoffs of visceral fat area for predicting MAFLD in China

**DOI:** 10.1186/s12944-022-01763-2

**Published:** 2022-12-31

**Authors:** Pingping Yu, Huachao Yang, Xiaoya Qi, Ruixue Bai, Shouqin Zhang, Jianping Gong, Ying Mei, Peng Hu

**Affiliations:** 1 Department of Health Management, The Second Affiliated Hospital of Chongqing Medical University, Chongqing, China; 2 Department of Infectious Diseases, Institute for Viral Hepatitis, The Key Laboratory of Molecular Biology for Infectious Diseases, Chinese Ministry of Education, The Second Affiliated Hospital of Chongqing Medical University, Chongqing, China; 3 Department of Hepatobiliary Surgery, The Second Affiliated Hospital of Chongqing Medical University, Chongqing, China

**Keywords:** Metabolic-associated fatty liver disease (MAFLD), Visceral fat area (VFA), Quantitative Computed Tomography (QCT)

## Abstract

**Background:**

Since the discovery of metabolic-associated fatty liver disease (MAFLD) in 2020, no report on the connection between the visceral fat area (VFA) and MAFLD has been published in China, and the ideal cutoffs of VFA for predicting MAFLD has not been determined so far. Thus, the purpose of this research was to clarify the relationship between VFA and MAFLD and the ideal cutoffs of VFA to predict MAFLD in the Chinese population.

**Methods:**

Five thousand three hundred forty subjects were included in this research, with 30% randomly selected for the validation set (*n *= 1602) and 70% for the Training set (*n* = 3738). The association between VFA and MAFLD was determined by multiple logistic regression. ROC curves were used to evaluate the prediction effect of VFA on MAFLD.

**Results:**

Multiple logistic regression analysis revealed that the VFA ORs (95% CIs) were 1.25 (1.20, 1.29) for women and 1.15 (1.12, 1.17) for men. Meanwhile, the VFA quartile OR (95% CI) were 3.07 (1.64, 5.75), 7.22 (3.97, 13.14), 18.91 (10.30, 34.71) for women and 3.07 (1.64, 5.75), 7.22 (3.97, 13.14),18.91 (10.30, 34.71) for men in the Q2, Q3, and Q4 groups compared with Q1. The ROC curve showed the VFA, WC, WHR, and WHtR to predict MAFLD, the AUC value of VFA was the highest and the prediction effect was the best. The ideal cutoffs of VFA to predict MAFLD was 115.55 cm^2^ for women and 178.35 cm^2^ for men, and the AUC was 0.788 and 0.795, respectively. Finally, the AUC was 0.773 for women and 0.800 for men in the validation set.

**Conclusion:**

VFA was an independent predictive factor for MAFLD, and the ideal cutoff of VFA to predict MAFLD was 115.55 cm^2^ in women and 178.35 cm^2^ in men.

**Supplementary Information:**

The online version contains supplementary material available at 10.1186/s12944-022-01763-2.

## Introduction

With improvements in living standards and changes in dietary structure and lifestyle, the prevalence and incidence of MAFLD associated with obesity/overweight, metabolic disorders, and type 2 diabetes (T2DM) have dramatically increased. Notably, the increased prevalence of MAFLD resulted in increased mortality from decompensated liver cirrhosis, hepatocellular carcinoma, and hepatic-related diseases [1, 2]. It is well documented that MAFLD can promote the development of cardiovascular and cerebrovascular diseases [3, 4], chronic kidney disease [5], ovarian syndrome[6], and malignant tumors (such as liver cancer, colorectal adenoma/adenocarcinoma, breast cancer, and lung adenocarcinoma) [7–10], seriously endangering human health and imposing a huge economic burden on society. MAFLD typically has an insidious onset and slow progression; the majority of patients manifest no obvious clinical symptoms or discomfort, which is easily overlooked. Therefore, it is crucial to find an early and non-invasive method for evaluation for MAFLD.

Abdominal obesity is considered to be one of the major risk factors for fatty liver. Quantitative indicators of abdominal obesity include visceral fat area (VFA), waist circumference (WC), waist-to-hip ratio (WHR), and waist-to-height ratio (WHtR) [1]. WC is currently recognized as the simplest and most practical index for measuring abdominal obesity [2]. WHR is the ratio of waist circumference to hip circumference and is an important indicator for determining central obesity. WHtR is the ratio of waist to height, which can reflect the accumulation of visceral fat. These indexes can't quantitatively reflect the degree of accumulation of abdominal. However, VFA is an accurate and reproducible indicator of abdominal obesity [11, 12], and it is a gold standard for the diagnosis of abdominal obesity, and it can accurately and visually reflect the accumulation of visceral fat and the distribution of fat [3]. Indeed, there is substantial evidence that VFA is closely related to T2DM [13], metabolic syndrome (MS) [14], and cardiovascular disease [15, 16]. Moreover, VFA is an independent risk factor for steatohepatitis with a dose–response relationship to its risk [17]. Excessive accumulation of visceral fat can increase fat deposition in the liver, promote the synthesis of triglycerides, release more free fatty acids into the blood, interfere with glucose metabolism, and lead to lipid metabolism disorders and insulin resistance, thereby promoting the formation of fatty liver [18, 19]. The accumulation of visceral fat may reduce adiponectin levels and release other inflammatory factors, triggering a series of metabolic disorders such as insulin resistance, inflammation, and fatty liver [20, 21]. Visceral adipose tissue can promote the secretion of large amounts of inflammatory cytokines for release, triggering inflammatory responses and oxidative stress, leading to increased expression of TNF-α, IL-1, IL-6, and other inflammatory cytokines, which contribute to accelerated liver damage and more rapid progression of fatty liver disease [22–25]. Therefore, measuring VFA is essential for evaluating patients with MAFLD.

Since MAFLD was proposed in 2020, there has been no report on the relationship between VFA and MAFLD, and the ideal cutoffs of VFA for predicting MAFLD have not been established in the Chinese population. In contrast, the VFA in the prediction of T2DM [13], cardiometabolic diseases [26], gastric cancer [27], and metabolic syndrome [28] have been established, and based on the results of previous studies, the cutoffs of VFA prediction for various diseases are also completely different. In addition, MAFLD varies significantly by gender, and its pathophysiological mechanisms are affected by gender and fat distribution [29], and thus, ignoring gender-specific analysis may mask crucial findings. Therefore, in this study, the VFA was taken as the breakthrough point to search for the association between VFA and MAFLD, and determine the ideal cutoffs of VFA to predict MAFLD in different genders.

## Materials and methods

### Study participants

This study collected people aged ≥ 18 years with QCT from July 1, 2020 to March 31, 2022 as research subjects. Exclusion criteria: (1) Incomplete or missing baseline data; (2) severe cardiac, hepatic, or renal insufficiency and (3) malignancy. To preserve the privacy of participants, untraceable codes were used to encode their identifiable information. The research was approved as Clinical Trial 2020 (261) by the Ethics Committee of the Second Affiliated Hospital of Chongqing Medical University, and all subjects signed an informed consent form.

### Laboratory and imaging evaluation

According to previous studies, risk factors affecting fatty liver were selected as covariates. The medical history included a history of hypertension, diabetes, smoking (with and without), alcohol consumption (no drinking, light drinking as defined as alcohol consumption < 140 g/week for men and < 70 g/week for women, and heavy drinking as defined as alcohol consumption ≥ 140 g/week for men and ≥ 70 g/week for women), and exercise (no exercise, 3 days/week or less, 3 days/week or more, and 30–60 min/day of moderate intensity exercise). The Omron body scale was used to check height and weight, Omron blood pressure monitor was used to measure systolic, diastolic, and heart rate. Blood tests included the levels of liver enzyme (γ-glutamyl transferase (GGT), albumin (ALB), 5 '- nucleosidase (5-NT), alanine aminotransferase (ALT), aspartic acid Aminotransferase (AST),) blood lipids (low-density lipoprotein-cholesterol (LDL-C), total cholesterol (TC), triglycerides (TG), and high-density lipoprotein-cholesterol (HDL-C)), renal function ((uric acid (UA), blood urea nitrogen (BUN), and serum creatinine (SCr)), fasting blood glucose (FPG), blood cell count ( platelet (PLT), white blood cell (WBC), and hemoglobin (HGB)), and HbA1c were measured with Hitachi automatic biochemical analyzer. Liver fat content (LFC) and VFA were measured using Quantitative Computed Tomography (QCT), a 64-slice CT scanner (model: SOMATOM go. Top) from Germany’s Siemens (the fourth-generation solid cylindrical membrane and image analysis system (QCT Pro 6.1) from Midways) was used, and scanning and computational processes were performed as described by Guo et al. [30].

### Diagnosis of MAFLD

As outlined by an international consensus statement of experts on the new definition of MAFLD, the diagnostic criteria were based on LFC ≥ 5% using QCT combined with one of the following three conditions: T2DM, BMI < 23 kg/m^2^ with ≥ 2 metabolic parameters, and overweight or obesity (defined as BMI ≥ 23 kg/m^2^ for the Asian population) [1]. Presence of at least two risk factors for metabolic abnormalities, including (1) Blood pressure ≥ 130/85 mmHg or under antihypertensive treatment; (2)Waist circumference (WC): men ≥ 90 cm and women ≥ 80 cm; (3) TG levels ≥ 1.70 mmol/L or under lipid-lowering therapy; (4) HDL-C levels < 1.0 mmol/L for men and < 1.3 mmol/L for women or on lipid-lowering therapy; (5) Pre-diabetes, FPG of 5.6 ~ 6.9 mmol/L or HbA1c of 5.7 ~ 6.4%. HOMA-IR and C-reactive protein levels were not determined. 

### Statistical analysis

Software packages R and EmpowerStats were used for data statistics and analysis for this study. Continuous variables are described by mean ± standard deviation (SD), categorical variables are expressed as percentages, The independent sample rank-sum test (Mann–Whitney test) was used for comparison between two groups of data with non-normal distribution, and independent samples T-test was used for comparison between two groups of data with normal distribution. Variables with variance inflation factor (VIF) values ≥ 5 were excluded by the independent variable collinearity stepwise screening method. Multiple logistic regression was used to clarify the relationship between VFA and MAFLD, and the receiver operating characteristics (ROC) curve was used to verify the predictive ability of VFA on MAFLD.

## Results

### Baseline characteristics of the study participants in the training set according to gender

**Fig. 1:**
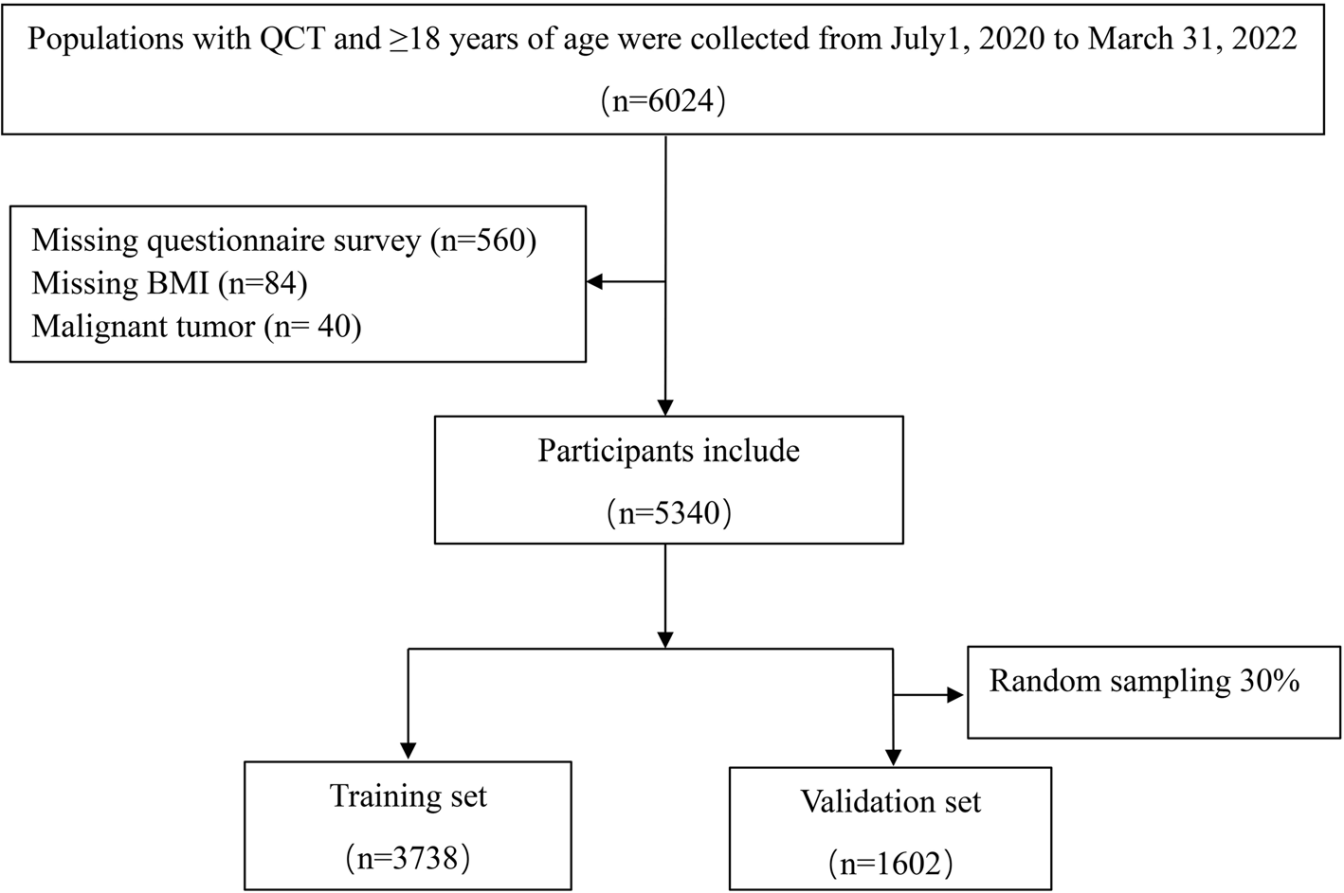
Study flowchart

A total of 6024 subjects were included, of whom 560 did not fill out the questionnaire, 84 had missing BMI data, and 40 had malignant tumors. Initially, 5340 subjects were included, and 30% of the subjects were randomly sampled as the internal validation set, with 3,738 subjects and 1,602 subjects in the training and the validation sets, respectively. (Fig. 1 Study flowchart). There was no statistical difference between the training set and validation set for all variables (Supplementary Table 1).

In the training set, the prevalence of MAFLD was 36.44% in women and 56.87% in men, and the prevalence of men is significantly higher than in women (χ^2^ = 154.39, *P* < 0.001). In the baseline data, women are older (*P* = 0.02) and have higher TC levels (*P* < 0.001)compared with men. While men had poorer metabolism, including higher BMI, higher blood pressure, higher WC, higher FPG levels, higher HbA1C levels, dyslipidemia (higher TG, lower HDL-C), higher UA levels, higher BUN levels, higher SCr levels, higher serum liver enzyme levels (GGT, ALT, AST), higher HGb, higher PLT, higher WBC, higher LFC, excess VFA, the above variables have statistical differences (*P* < 0.001). Men had higher proportions of hypertensive patients (χ^2^ = 5.49, *P* = 0.02), higher proportions of diabetics patients (χ^2^ = 30.50, *P* < 0.001), higher proportions of smokers (χ^2^ = 857.07, *P* < 0.001), and higher proportions of alcohol consumers (χ^2^ = 927.93, *P* < 0.001). There were no statistical differences in LDL-C levels, 5-NT levels, and physical activity between the two groups in the training set (Table 1) .

**Table 1:** Baseline characteristics of study populations by gender in the training set

Variable	Women (*n* = 1663)	Men (*n* = 2075)	*P*-value
**MAFLD**			< 0.001
No	1057 (63.56%)	895 (43.13%)	
Yes	606 (36.44%)	1180 (56.87%)	
**Age, year**	53.66 ± 10.27	52.91 ± 9.46	0.020
**BMI, kg/m**^**2**^	23.22 ± 2.97	24.83 ± 2.88	< 0.001
**SBP, mmHg**	124.17 ± 18.90	127.22 ± 17.38	< 0.001
**DBP, mmHg**	72.19 ± 10.57	77.81 ± 11.25	< 0.001
**WC, CM**	75.99 ± 7.79	86.65 ± 7.83	< 0.001
**WHR**	0.82 ± 0.06	0.90 ± 0.06	< 0.001
**WHtR**	0.49 ± 0.05	0.52 ± 0.05	< 0.001
**GGT, U/L**	23.46 ± 20.29	39.87 ± 38.04	< 0.001
**ALT, U/L**	19.38 ± 12.13	26.16 ± 16.96	< 0.001
**AST, U/L**	21.14 ± 7.24	23.09 ± 10.44	< 0.001
**5-NT, U/L**	4.12 ± 2.14	4.06 ± 1.98	0.434
**ALB, g/L**	43.24 ± 9.32	45.27 ± 6.56	< 0.001
**UA, mg/dL**	306.72 ± 69.62	405.21 ± 89.97	< 0.001
**BUN, umol/L**	5.40 ± 1.33	5.83 ± 1.40	< 0.001
**SCr, umol/L**	54.91 ± 12.64	76.03 ± 15.54	< 0.001
**TG, mmol/L**	1.52 ± 1.09	2.19 ± 2.23	< 0.001
**TC, mmol/L**	5.37 ± 0.98	5.15 ± 1.05	< 0.001
**HDL-C, mmol/L**	1.49 ± 0.33	1.22 ± 0.28	< 0.001
**LDL-C, mmol/L**	2.87 ± 0.76	2.89 ± 0.76	0.584
**FPG, mmol/L**	5.17 ± 0.97	5.58 ± 1.72	< 0.001
**HbA1C, %**	5.71 ± 0.82	5.91 ± 1.09	< 0.001
**HB, g/L**	133.38 ± 11.08	153.77 ± 11.98	< 0.001
**PLT, 10**^**9**^**/L**	238.98 ± 62.59	222.29 ± 64.20	< 0.001
**WBC, 109/L**	5.67 ± 1.42	6.35 ± 1.67	< 0.001
**LFC, %**	6.26 ± 4.67	7.52 ± 5.53	< 0.001
**VFA, cm**^**2**^	106.65 ± 49.55	193.99 ± 75.43	< 0.001
**Virus hepatitis**			0.533
No	1622 (97.5%)	2017 (97.2%)	
Yes	41 (2.5%)	58 (2.8%)	
**Hypertension**			0.019
No	1494 (89.84%)	1813 (87.37%)	
Yes	169 (10.16%)	262 (12.63%)	
**Diabetes**			< 0.001
No	1610 (96.81%)	1923 (92.67%)	
Yes	53 (3.19%)	152 (7.33%)	
**Smoking**			< 0.001
No	1642 (98.74%)	1193 (57.49%)	
Yes	21 (1.26%)	882 (42.51%)	
**Drinking**			< 0.001
No	1564 (94.05%)	930 (44.82%)	
Light drinking	97 (5.83%)	1045 (50.36%)	
Heavy drinking	2 (0.12%)	100 (4.82%)	
**Physical activity**			0.129
Low	564 (33.96%)	675 (32.58%)	
Moderate	586 (35.28%)	695 (33.54%)	
High	511 (30.76%)	702 (33.88%)	

Data were presented as mean (SD) or n (%)

Continuous variables were represented as mean ± SD; categorical variables were expressed as numbers (percentages); the Kruskal–Wallis rank test was used for continuous variables and the chi-square test for categorical variables, and when the expected value was < 10, the Fisher’s exact test was used

*BMI* Body mass index, *WC* Waist circumference, *WHR* Waist-to-hip ratio, *WHtR* Waist-to-Height ratio, *SBP* Systolic pressure, *DBP* Diastolic pressure, *GGT* γ-glutamyl transpeptidase, *ALT* Alanine aminotransferase, *AST* Aspartate aminotransferase, *5-NT* 5'- nucleotidase, *ALB* Albumin, *UA* Uric acid, *BUN* Blood urea nitrogen, *SCr* Serum creatinine, *TG* Triglyceride, *TC* Total cholesterol, *HDL-C* High-density lipoprotein cholesterol, *LDL-C* Low-density lipoprotein cholesterol, *FPG* Fasting blood glucose, *HbA1c* Glycosylated hemoglobin, *HGB* hemoglobin, *PLT* Platelet *WBC* White blood cells, *LFC* Liver fat content, *VFA* Visceral fat area

### Comparing differences in clinical parameters between Non-MAFLD and MAFLD groups of different genders

In the training set, patients with MAFLD were older, had higher BMI, higher WC, higher serum liver enzyme levels (GGT, ALT, AST, 5NT), higher UA levels, higher TG levels, lower HDL-C levels, higher FPG levels, and HbA1C levels, higher WBC levels, higher LFC, higher VFA compared with non-MAFLD groups in men and women, the above variables have statistical differences (*P* < 0.05). At the same time, patients with MAFLD had higher proportions of hypertension patients (χ^2^ = 125.44, *P* < 0.001; χ^2^ = 39.35, *P* < 0.001) and higher proportions of diabetes patients (χ^2^ = 47.21, *P* < 0.001; χ^2^ = 27.03, *P* < 0.001) compared with Non-MAFLD groups in men and women. Moreover, MAFLD had higher PLT (*P* < 0.001), higher ALB (*P* = 0.044), and higher BUN (*P* = 0.006) in women, but the difference was not statistically significant in men. Patients with MAFLD had higher LDL-C (*P* = 0.043) and HB (*P* = 0.001) in men, while there was no statistical difference in women. There was no statistical difference in SCr levels, TC levels, smoking and drinking, and physical activity between non-MAFLD and MAFLD groups in men and women (Table 2).

**Table 2:** Compare the differences in clinical parameters of Non-MAFLD and MAFLD in different gender

	**Women**			**Men**		
**Variable**	**Non-MAFLD (*n* = 1057)**	**MAFLD (*n* = 606)**	***P*-value**	**Non-MAFLD (*n *= 895)**	**MAFLD (*n* = 1180)**	***P*-value**
**Age, year**	51.95 ± 9.66	56.65 ± 10.62	< 0.001	52.15 ± 9.18	53.48 ± 9.64	0.001
**BMI, kg/m**^**2**^	22.14 ± 2.40	25.11 ± 2.94	< 0.001	23.40 ± 2.59	25.92 ± 2.59	< 0.001
**SBP, mmHg**	120.03 ± 16.58	131.38 ± 20.48	< 0.001	123.13 ± 16.40	130.32 ± 17.46	< 0.001
**DBP, mmHg**	70.57 ± 10.26	75.01 ± 10.51	< 0.001	75.33 ± 10.53	79.70 ± 11.41	< 0.001
**WC, CM**	73.28 ± 6.59	80.72 ± 7.46	< 0.001	82.84 ± 7.31	89.54 ± 6.92	< 0.001
**WHR**	0.81 ± 0.06	0.86 ± 0.06	< 0.001	0.88 ± 0.05	0.92 ± 0.05	< 0.001
**WHtR**	0.47 ± 0.05	0.52 ± 0.05	< 0.001	0.49 ± 0.04	0.53 ± 0.04	< 0.001
**GGT, U/L**	20.58 ± 18.85	28.32 ± 21.66	< 0.001	33.74 ± 32.37	44.48 ± 41.22	< 0.001
**ALT, U/L**	17.50 ± 11.39	22.56 ± 12.69	< 0.001	22.84 ± 16.80	28.66 ± 16.65	< 0.001
**AST, U/L**	20.52 ± 7.03	22.18 ± 7.47	< 0.001	22.21 ± 8.41	23.76 ± 11.71	< 0.001
**5-NT, U/L**	3.88 ± 2.13	4.59 ± 2.08	< 0.001	3.74 ± 1.59	4.32 ± 2.22	< 0.001
**ALB, g/L**	42.89 ± 10.31	43.85 ± 7.26	0.044	45.22 ± 7.07	45.31 ± 6.15	0.756
**UA, mg/dL**	291.12 ± 61.25	333.09 ± 74.83	< 0.001	389.21 ± 79.78	417.39 ± 95.26	< 0.001
**BUN, umol/L**	5.33 ± 1.28	5.52 ± 1.41	0.006	5.79 ± 1.37	5.86 ± 1.42	0.290
**SCr, umol/L**	55.09 ± 11.07	54.61 ± 14.93	0.458	75.84 ± 13.79	76.17 ± 16.76	0.634
**TG, mmol/L**	1.24 ± 0.60	1.99 ± 1.49	< 0.001	1.68 ± 1.17	2.57 ± 2.71	< 0.001
**TC, mmol/L**	5.40 ± 0.97	5.33 ± 1.02	0.185	5.11 ± 0.92	5.19 ± 1.13	0.090
**HDL-C, mmol/L**	1.57 ± 0.33	1.35 ± 0.29	< 0.001	1.28 ± 0.29	1.17 ± 0.27	< 0.001
**LDL-C, mmol/L**	2.85 ± 0.72	2.91 ± 0.81	0.095	2.85 ± 0.72	2.92 ± 0.79	0.043
**FPG, mmol/L**	4.96 ± 0.60	5.51 ± 1.32	< 0.001	5.28 ± 1.48	5.80 ± 1.86	< 0.001
**HbA1C, %**	5.50 ± 0.71	5.94 ± 0.87	< 0.001	5.69 ± 0.93	6.05 ± 1.15	< 0.001
**HGB, g/L**	133.16 ± 10.61	133.76 ± 11.82	0.304	152.77 ± 12.49	154.53 ± 11.52	0.001
**PLT, 10**^**9**^**/L**	234.94 ± 60.26	245.85 ± 65.84	< 0.001	219.70 ± 62.57	224.22 ± 65.35	0.118
**WBC, 109/L**	5.45 ± 1.36	6.03 ± 1.44	< 0.001	6.02 ± 1.57	6.60 ± 1.70	< 0.001
**LFC, %**	4.11 ± 2.60	10.02 ± 5.08	< 0.001	3.66 ± 2.67	10.45 ± 5.34	< 0.001
**VFA, cm**^**2**^	89.07 ± 42.27	137.19 ± 46.37	< 0.001	155.21 ± 71.28	223.38 ± 64.42	< 0.001
**Virus hepatitis**			0.105			0.996
No	1026 (97.1%)	596 (98.3%)		870 (97.2%)	1147 (97.2%)	
Yes	31 (2.9%)	10 (1.7%)		25 (2.8%)	33 (2.8%)	
**Hypertension**			< 0.001			< 0.001
No	1016 (96.12%)	478 (78.88%)		829 (92.63%)	984 (83.39%)	
Yes	41 (3.88%)	128 (21.12%)		66 (7.37%)	196 (16.61%)	
**Diabetes**			< 0.001			< 0.001
No	1047 (99.05%)	563 (92.90%)		860 (96.09%)	1063 (90.08%)	
Yes	10 (0.95%)	43 (7.10%)		35 (3.91%)	117 (9.92%)	
**Smoking**			0.226			0.898
No	1041 (98.49%)	601 (99.17%)		516 (57.65%)	677 (57.37%)	
Yes	16 (1.51%)	5 (0.83%)		379 (42.35%)	503 (42.63%)	
**Drinking**			0.357			0.836
No	989 (93.57%)	575 (94.88%)		407 (45.47%)	523 (44.32%)	
Light drinking	66 (6.24%)	31 (5.12%)		444 (49.61%)	601 (50.93%)	
Heavy drinking	2 (0.19%)	0 (0.00%)		44 (4.92%)	56 (4.75%)	
**Physical activity**			0.066			0.137
Low	339 (32.13%)	225 (37.13%)		271 (30.31%)	404 (34.30%)	
Moderate	391 (37.06%)	195 (32.18%)		315 (35.23%)	380 (32.26%)	
High	325 (30.81%)	186 (30.69%)		308 (34.45%)	394 (33.45%)	

Data were presented as mean (SD) or n (%)

### VFA is an independent risk factor for MAFLD

Multiple logistic regression was used to clarify the correlation between VFA and MAFLD in different models (Table 3). Variables WC and TC were excluded by independent variable collinearity stepwise screening. Multiple logistic regression analysis showed an ORs (95% CIs) of 1.27 (1.24 − 1.30), 1.25 (1.21 − 1.28), 1.25 (1.20 − 1.29) for women and 1.16 (1.15 − 1.18), 1.16 (1.15–1.18), 1.15 (1.12–1.17) for men in the unadjusted, minimally adjusted, and fully adjusted models, respectively. After adjusting for confounders, quartile analysis of VFA to clarify the relationship between VFA and MAFLD, and found that the ORs (95% CIs) of MAFLD risk in groups Q2, Q3, and Q4 compared with the lowest VFA group (Q1) was 3.77 (2.77- 5.14), 7.83 (5.20–11.79), and 21.23 (8.16–55.25) for women and 3.07 (1.64–5.75), 7.22 (3.97–13.14), 18.91 (10.30–34.71) for men in the fully adjusted model, respectively. The risk of MAFLD increased with increasing VFA, with a trend test *P* value of < 0.001, suggesting a statistically significant increase.

**Table 3:** Relationship between VFA and MAFLD in different models by multiple logistic regression

	Women			Men		
Exposure	Non-adjusted model OR, 95%CI, *P*	Minimally-adjusted model OR, 95%CI, *P*	Fully-adjusted model OR, 95%CI, *P*	Non-adjusted model OR, 95%CI, *P*	Minimally-adjusted model OR, 95%CI, *P*	Fully-adjusted model OR, 95%CI, *P*
VFA	1.27(1.24–1.30) < 0.001	1.25(1.21–1.28) < 0.001	1.25(1.20–1.29) < 0.001	1.16(1.15–1.18) < 0.001	1.16(1.15–1.18) < 0.001	1.15(1.12–1.17) < 0.001
VFA (quartile)						
Q1	Referent	Referent	Referent	Referent	Referent	Referent
Q2	4.40 (3.51–5.53) < 0.001	3.90 (3.09- 4.93) < 0.001	3.77 (2.77–5.14) < 0.001	2.86(1.78–4.59) < 0.001	2.84(1.77–4.56) < 0.001	3.07(1.64–5.75) 0.001
Q3	11.59 (8.58–15.66) < 0.001	9.59 (7.04–13.08) < 0.001	7.83 (5.20–11.79) < 0.001	7.37(4.73–11.48) < 0.001	7.27(4.67–11.33) < 0.001	7.22(3.97–13.14) < 0.001
Q4	29.73 (14.86–59.47) < 0.001	23.06 (11.43–46.52) < 0.001	21.23 (8.16–55.25) < 0.001	22.14 (14.18–34.55) < 0.001	21.71 (13.90–33.90) < 0.001	18.91 (10.30–34.71) < 0.001
*P* for trend	< 0.001	< 0.001	< 0.001	< 0.001	< 0.001	< 0.001

Non-adjusted model: None

Minimally-adjusted model: Gender and Age

Fully-adjusted model: Gender, Age, SBP, DBP, GGT, ALT, AST, 5NT, UA, BUN, SCr, LDL-C, HDL-C, HB, PLT, WBC, FPG levels, HbA1C, Virus hepatitis, Hypertension, Diabetes, Smoking, Drinking, and Physical activity. The continuous variable VFA was processed according to four categorical variables (Q1-Q4), and then multiple logistic regression analysis was carried out

### Comparison of VFA, WC, WHtR, and WHR for predicting MAFLD risk

**Fig. 2:**
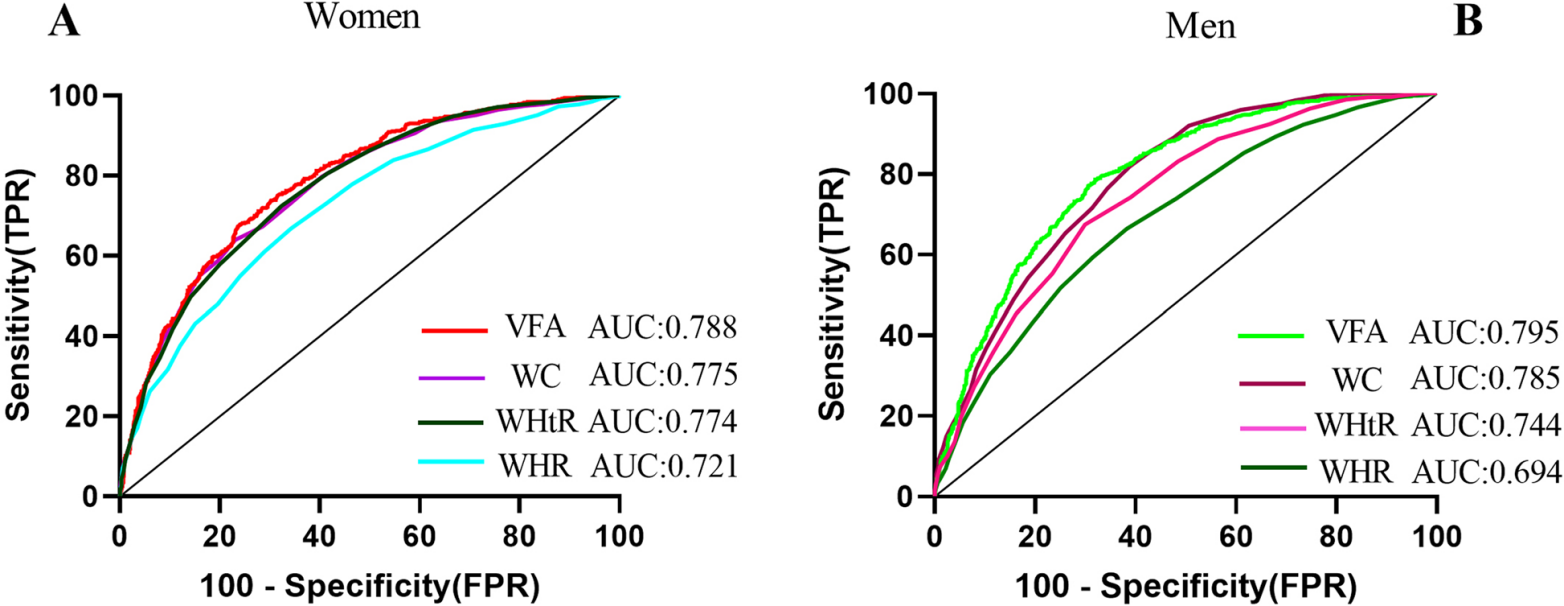
The ROC curves for VFA, WC, WHR, and WHtR for predicting MAFLD risk for men and women

The ROC curve shows that VFA, WC, WHtR, and WHR predict MAFLD in different genders (Fig. 2). The AUC(95CI) of VFA, WC, WHR, and WHtR in women is 0.788 (0.766–0.810), 0.775 (0.752–0.798), 0.721 (0.696–0.747) and 0.774 (0.751–0.797), respectively; The AUC(95CI) of VFA, WC, WHR and WHtR in men is 0.795 (0.777–0.814), 0.785 (0.766–0.804), 0.694 (0.671–0.717) and 0.744 (0.723–0.766), respectively; The AUC values of VFA, WC, and WHTR variables were greater than 0.7, indicating that VFA, WC, and WHTR had certain predictive value for MAFLD risk in different genders, among which VFA has the largest AUC and the greatest predictive value for MAFLD.

### The ideal cutoffs of VFA for the prediction of MAFLD risk

**Fig. 3:**
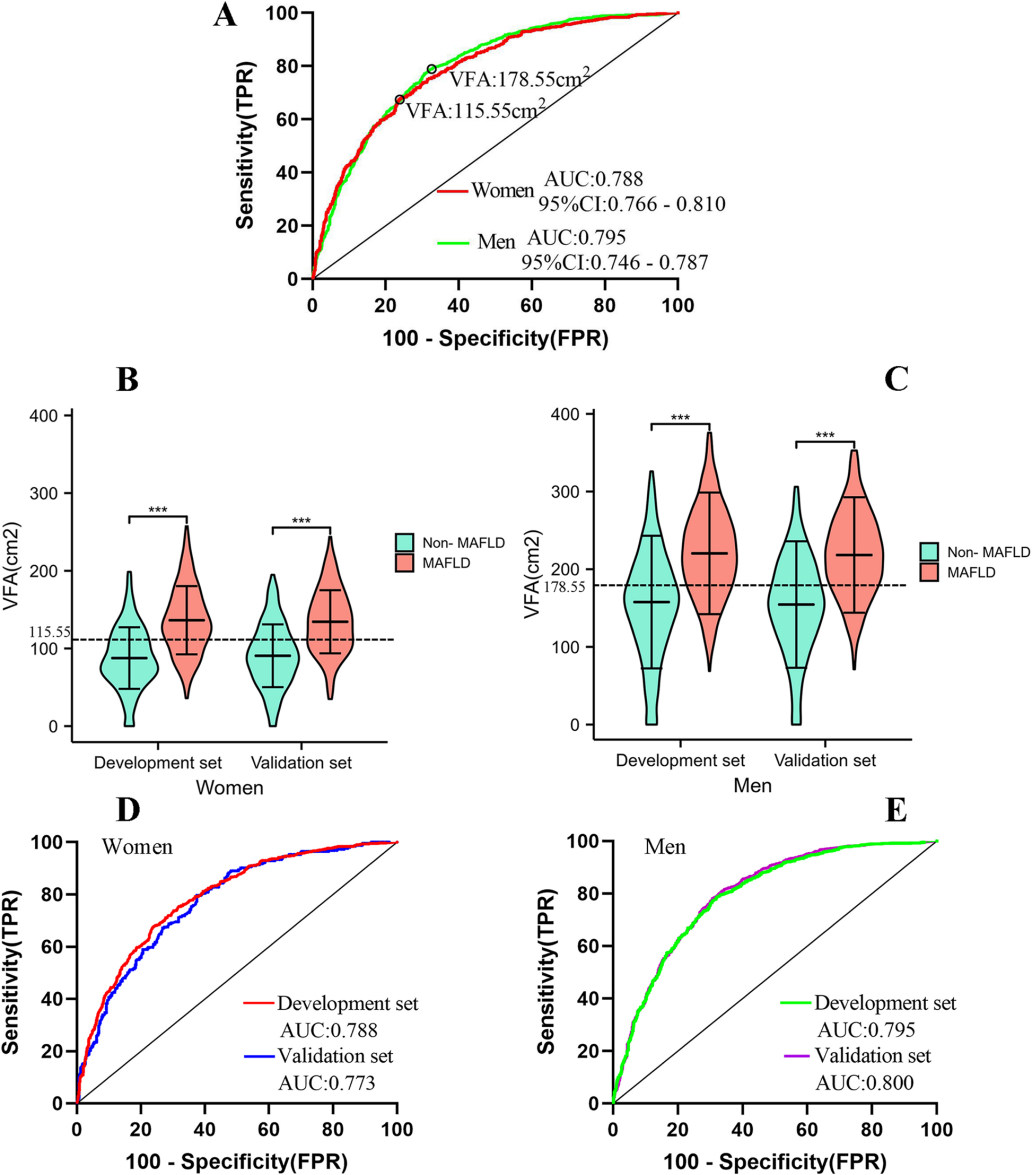
Receiver operating characteristic curve of VFA for predicting incident MAFLD in women and men **A**. The violin chart reflects the distribution and probability density of different genders of VFA for participants with and without MAFLD in the training set and validation set **B**, **C**. The ROC curve was used for risk prediction evaluation in the training and validation sets **D**, **E**. AUC, area under the curve; CI, confidence interval

ROC curves were used to assess the ability of VFA to predict MAFLD and the ideal cutoffs of VFA for predicting MAFLD in different genders. The results demonstrated that the AUC (95% CI) of VFAs was 0.788 (0.766–0.81) in women and 0.795 (0.777 -0.814) in men. The specificity and sensitivity of VFA in predicting MAFLD were 0.760 and 0.678 in women, and 0.644 and 0.772 in men, respectively. Furthermore, the ideal cutoff of VFA was 115.55 cm^2^ for women and 178.55 cm^2^ for men (Fig. 3A). As expected, there was a significant gender difference in the cutoffs of VFA for predicting MAFLD, and men had higher cutoffs in VFA than women. The violin chart reflects the distribution and probability density of the different gender of VFA for patients with and without MAFLD in the training and validation sets (Figs. 3B, C). The results showed that the AUCs of VFAs were 0.788 and 0.773 for women and 0.795 and 0.800 for men in the training set and validation set, respectively. (Figs. 3D, E). VFA could accurately predict the risk of MAFLD, thus providing a clinical basis for the prevention and treatment of MAFLD patients.

## Discussion

The results of this study signaled that the MAFLD population was older, with higher liver enzymes, poorer metabolism, higher LFC, and higher VFA than non-MAFLD. Regression analysis showed that VFA was an independent predictor of MAFLD, and its risk increases with increasing VFA quartiles. The ROC curve showed the VFA, WC, WHR, and WHtR to predict MAFLD in different genders, the results showed that VFA is a better predictor of MAFLD risk than WC, WHR, and WHtR. Interestingly, the study found a significant difference in gender in the VFA prediction of MAFLD risk, the ideal cutoffs of VFA were 115.55 cm^2^ for women and 178.35 cm^2^ for men. Further through internal validation, the results showed that the AUC of the VFA was very close and the AUC was greater than 0.75 in the validation and training sets, which implies that the VFA has good distinguishing ability and accuracy in predicting MAFLD risks.

This study highlights the importance of VFA for the risk assessment of MAFLD. Although the status of BMI and WC in risk prediction has been established. BMI is affected by bones and muscle, cannot reflect the fat content and body fat distribution, and cannot effectively reflect the abdominal and visceral fat accumulation. Individuals with normal or low BMI may have visceral fat accumulation. WC cannot distinguish visceral fat from subcutaneous fat and is influenced by height [31], and thus it cannot accurately predict the risk of MAFLD. In recent years, the role of VFA in a variety of chronic diseases has attracted attention. VFA is considered a more precise indicator of abdominal obesity and metabolic risk factors than BMI and WC [28]. Studies have established that VFA is closely related to the severity of hepatic steatosis [32] and is also a strong predictor of NAFLD [33]. Compared with subcutaneous fat, VFA was more closely related to metabolic abnormalities [34]. Therefore, VFA can become an essential indicator for predicting the risk of MAFLD.

Obesity and abdominal obesity defined by BMI and WC have different cutoffs in different races and regions, and the cutoffs of VFA to predict different diseases also differ. A study has shown that VFA is closely related to MS, and the ideal cutoff for VFA was 81.1 cm^2^ of women and 84.7 cm^2^ of men to predict MS in T2DM [35]. 4.8-year follow-up result showed that VFA was an independent predictor for T2DM, the prediction of T2DM with VFA ≥ 130 cm^2^ in men and ≥ 85 cm^2^ in women in South Korea [13]. Likewise, another study determined that the ideal cutoffs for VFA were 120 cm^2^ of men and 80 cm^2^ of women to predict T2DM in South Korea [36], the VFA for predicting T2DM in the above two studies is different, which may be attributed to different included populations and independent variables. Meanwhile, the VFA was 134.6 cm^2^ in men and 91.1 cm^2^ in women to predict MS in Korean adults [28]. A Turkish study has shown that pancreatic steatosis is associated with high VFA, VFA ≥ 107.2 cm^2^ could predict pancreatic steatosis [37].

Recent studies have demonstrated that the cutoffs of VFA for predicting NAFLD or MAFLD are significantly different among different ethnic groups and regions. In a study in Taiwan, the ideal cutoff for VFA to predict NAFLD was 70.5 cm^2^ [33]. In another study, the ideal cutoffs for VAR predicted NAFLD to be 3.469 for men and 6.357 for women in Tianjin [38]. Studies have demonstrated that decreased muscle mass and increased visceral fat exacerbate the increased risk of NAFLD in Japan [39]. Cho et al. study described that the low-grade skeletal muscle mass to visible fat area ratio was an independent risk factor for NAFLD in Korean [40]. Another study in South Korea showed that the ideal cutoffs for VFA to identify with lean NAFLD was 50.2 cm^2^ for men and 40.5 cm^2^ for women and to identify with overweight or obese NAFLD was 100.6 cm^2^ for men and 68.0 cm2 for women [41]. Sogabe et al. compared gender differences in alcohol consumption and abdominal fat between NAFLD and MAFLD in the Japanese population and evinced that the cutoffs for VFA to identify NAFLD and MAFLD were 108.1 ± 34.1 and 140.7 ± 46.0 for men, and 96.4 ± 27.8 and 120.8 ± 42.7 for women, respectively, the diagnostic criteria for NAFLD and MAFLD are different, and the cutoffs for VFA to predict NAFLD and MAFLD are different, this study also validated this point [42]. The prevalence and severity of fatty liver vary significantly among populations, and these differences can be attributed to several factors, including region, ethnicity, lifestyle, metabolic complications, and environmental and genetic epigenetic factors [43–47]. The new MAFLD definition has been endorsed by more than 1000 signatories from 134 countries and is advocated, given that it more accurately reflects the potential pathogenesis than NAFLD [48]. Therefore, the application of the definition and diagnostic criteria of MAFLD in this study is more conducive to the intervention and management of fatty liver.

### Strength and study limitations

To the best of our knowledge, this is the first time that VFA is an independent predictor of MAFLD, and the ideal cutoffs of VFA to predict the risk of MAFLD are determined in the Chinese population. The results of this study revealed that there are gender-specific at the ideal cutoffs of VFA for predicting the risk of MAFLD, which can facilitate its early diagnosis and treatment. However, this study has several limitations. To begin, considering that this was a single-center, cross-sectional study, the results may not be generalizable to a global population. Secondly, observational studies can only prove an association between VFA and MAFLD risk but cannot provide definitive conclusions about causality. Thirdly, subgroup analysis was not performed herein. Therefore, large-scale, multi-center studies should be conducted in the future to analyze the relationship between VFA and MAFLD in different subgroups according to BMI, diagnostic criteria of MAFLD, HBV infection, alcohol consumption, and metabolism.

To conclude, this is the first time that VFA is an independent predictor of MAFLD in China. There was a significant gender difference in the ideal cutoffs for VFA to predict MAFLD risk, which was 115.55 cm^2^ for women and 178.55 cm^2^ for men. Therefore, regular testing of VFAs is strongly recommended for the early prediction of the risk of MAFLD to better guide lifestyle interventions and health management.

## Supplementary Information

**Additional file 1: Supplementary Table 1.** Comparison of baseline clinical data for patients in the training set and validation set.

## Data Availability

The datasets generated and analyzed in this study are available from Dr. Pingping Yu upon reasonable request.

## References

[1] Eslam M, Newsome PN, Sarin SK (2020). A new definition for metabolic dysfunction-associated fatty liver disease: an international expert consensus statement. J Hepatol.

[2] Eslam M, Sarin SK, Wong VW (2020). The Asian Pacific Association for the Study of the Liver clinical practice guidelines for the diagnosis and management of metabolic associated fatty liver disease. Hepatol Int.

[3] Kim H, Lee CJ, Ahn SH (2022). MAFLD predicts the risk of cardiovascular disease better than NAFLD in asymptomatic subjects with health check-ups. Dig Dis Sci.

[4] Henson JB, Simon TG, Kaplan A (2020). Advanced fibrosis is associated with incident cardiovascular disease in patients with non-alcoholic fatty liver disease. Aliment Pharmacol Ther.

[5] Sun DQ, Jin Y, Wang TY, et al. MAFLD and risk of CKD. Metabol. 2021;115:154433. 10.1016/j.metabol.2020.154433 33212070

[6] Asfari MM, Sarmini MT, Baidoun F, et al. Association of non-alcoholic fatty liver disease and polycystic ovarian syndrome. BMJ Open Gastroenterol. 2020;7(1):e000352. 10.1136/bmjgast-2019-000352 PMC7418668 32784205

[7] Kim GA, Lee HC, Choe J (2018). Association between non-alcoholic fatty liver disease and cancer incidence rate. J Hepatol.

[8] Allen AM, Hicks SB, Mara KC (2019). The risk of incident extrahepatic cancers is higher in non-alcoholic fatty liver disease than obesity - a longitudinal cohort study. J Hepatol.

[9] Fukunaga S, Nakano D, Kawaguchi T (2021). Non-obese MAFLD is associated with colorectal adenoma in health check examinees: a multicenter retrospective study. Int J Mol Sci.

[10] Kwak MS, Yim JY, Yi A, Chung GE (2019). Nonalcoholic fatty liver disease is associated with breast cancer in nonobese women. Dig Liver Dis.

[11] Kouvari M, Polyzos SA, Chrysohoou C (2022). Skeletal muscle mass and abdominal obesity are independent predictors of hepatic steatosis and interact to predict ten-year cardiovascular disease incidence: data from the ATTICA cohort study. Clin Nutr.

[12] ZZhao D, Cui H, Shao Z, Cao L. Abdominal obesity, chronic inflammation and the risk of non-alcoholic fatty liver disease. Ann Hepatol. 2022:100726. 10.1016/j.aohep.2022.100726 35636732

[13] Kim EH, Kim HK, Lee MJ (2022). Sex differences of visceral fat area and visceral-to-subcutaneous fat ratio for the risk of incident type 2 diabetes mellitus. Diabetes Metab J.

[14] Jeon HH, Lee YK, Kim DH (2021). Risk for metabolic syndrome in the population with visceral fat area measured by bioelectrical impedance analysis. Korean J Intern Med.

[15] Tanaka T, Kishi S, Ninomiya K (2019). Impact of abdominal fat distribution, visceral fat, and subcutaneous fat on coronary plaque scores assessed by 320-row computed tomography coronary angiography. Atherosclerosis.

[16] Rodriguez-Granillo GA, Reynoso E, Capunay C (2018). Pericardial and visceral, but not total body fat, are related to global coronary and extra-coronary atherosclerotic plaque burden. Int J Cardiol.

[17] Vural Keskinler M, Mutlu HH, Sirin A (2021). Visceral adiposity index as a practical tool in patients with biopsy-proven nonalcoholic fatty liver disease/nonalcoholic steatohepatitis. Metab Syndr Relat Disord.

[18] Antonio-Villa NE, Bello-Chavolla OY, Vargas-Vázquez A (2021). Increased visceral fat accumulation modifies the effect of insulin resistance on arterial stiffness and hypertension risk. Nutr Metab Cardiovasc Dis.

[19] Ghosh A, Gao L, Thakur A (2017). Role of free fatty acids in endothelial dysfunction. J Biomed Sci.

[20] MoonHyun Uk, HaKyoung Hwa, HanSeung Jin, et al. The association of adiponectin and visceral fat with insulin resistance and β-Cell dysfunction. J Korean Med Sci. 2018;34(1):e7. 10.3346/jkms.2019.34.e7 PMC6318440 30618514

[21] Mavilia MG, Wu GY (2021). Liver and serum adiponectin levels in non-alcoholic fatty liver disease. J Dig Dis.

[22] Boutari C, Perakakis N, Mantzoros CS (2018). Association of adipokines with development and progression of nonalcoholic fatty liver disease. Endocrinol Metab (Seoul).

[23] Masarone M, Rosato V, Dallio M, et al. Role of oxidative stress in pathophysiology of nonalcoholic fatty liver disease. Oxid Med Cell Longev. 2018;2018:9547613. 10.1155/2018/9547613 PMC6016172 29991976

[24] Perumpail BJ, Khan MA, Yoo ER (2017). Clinical epidemiology and disease burden of nonalcoholic fatty liver disease. World J Gastroenterol.

[25] Abenavoli L, Peta V (2014). Role of adipokines and cytokines in non-alcoholic fatty liver disease. Rev Recent Clin Trials.

[26] Polcrova A, Pavlovska I, Maranhao Neto GA (2021). Visceral fat area and cardiometabolic risk: The Kardiovize study. Obes Res Clin Pract.

[27] Gao B, Liu Y, Ding C (2020). Comparison of visceral fat area measured by CT and bioelectrical impedance analysis in Chinese patients with gastric cancer: a cross-sectional study. BMJ Open.

[28] Lee A, Kim YJ, Oh SW (2018). Cut-off values for visceral fat area identifying korean adults at risk for metabolic syndrome. Korean J Fam Med.

[29] Wong VW, Wong GL, Woo J (2021). Impact of the new definition of metabolic associated fatty liver disease on the epidemiology of the disease. Clin Gastroenterol Hepatol.

[30] Guo Z, Blake GM, Li K (2020). Liver fat content measurement with quantitative CT validated against MRI proton density fat fraction: a prospective study of 400 healthy volunteers. Radiol.

[31] Schneider HJ, Friedrich N, Klotsche J (2010). The predictive value of different measures of obesity for incident cardiovascular events and mortality. J Clin Endocrinol Metab.

[32] Choi MH, Choi JI, Park MY (2018). Validation of intimate correlation between visceral fat and hepatic steatosis: Quantitative measurement techniques using CT for area of fat and MR for hepatic steatosis. Clin Nutr.

[33] Ko YH, Wong TC, Hsu YY (2017). The correlation between body fat, visceral fat, and nonalcoholic fatty liver disease. Metab Syndr Relat Disord.

[34] Shah RV, Murthy VL, Abbasi SA (2014). Visceral adiposity and the risk of metabolic syndrome across body mass index: the MESA Study. JACC Cardiovasc Imaging.

[35] Yang X, Lin Y, Xu GD (2021). Optimal cut-off values of visceral fat area for predicting metabolic syndrome among type 2 diabetes patients in Ningbo. China Diabetes Metab Syndr Obes.

[36] Kim EH, Kim HK, Bae SJ (2018). Gender differences of visceral fat area for predicting incident type 2 diabetes in Koreans. Diabetes Res Clin Pract.

[37] Gursoy Coruh A, Uzun C, Akkaya Z, Halil EA (2020). The relation of CT quantified pancreatic fat index with visceral adiposity and hepatic steatosis. Turk J Surg.

[38] Shi YX, Chen XY, Qiu HN (2021). Visceral fat area to appendicular muscle mass ratio as a predictor for nonalcoholic fatty liver disease independent of obesity. Scand J Gastroenterol.

[39] Shida T, Akiyama K, Oh S (2018). Skeletal muscle mass to visceral fat area ratio is an important determinant affecting hepatic conditions of non-alcoholic fatty liver disease. J Gastroenterol.

[40] Cho Y, Chang Y, Ryu S (2022). Skeletal muscle mass to visceral fat area ratio as a predictor of NAFLD in lean and overweight men and women with effect modification by sex. Hepatol Commun.

[41] Lee S, Kim KW, Lee J (2022). Sex-specific cutoff values of visceral fat area for lean vs. overweight/obese nonalcoholic fatty liver disease in Asians. J Clin Transl Hepatol..

[42] Sogabe M, Okahisa T, Kurihara T (2022). Comparison of the role of alcohol consumption and qualitative abdominal fat on NAFLD and MAFLD in males and females. Sci Rep.

[43] Younossi Z, Anstee QM, Marietti M (2018). Global burden of NAFLD and NASH: trends, predictions, risk factors and prevention. Nat Rev Gastroenterol Hepatol.

[44] Wong VW, Wong GL, Yeung DK (2015). Incidence of non-alcoholic fatty liver disease in Hong Kong: a population study with paired proton-magnetic resonance spectroscopy. J Hepatol.

[45] Yi M, Chen RP, Yang R (2017). Increased prevalence and risk of non-alcoholic fatty liver disease in overweight and obese patients with type 2 diabetes in South China. Diabet Med.

[46] Wesolowski SR, Kasmi KC, Jonscher KR (2017). Developmental origins of NAFLD: a womb with a clue. Nat Rev Gastroenterol Hepatol.

[47] Yu Y, Cai J, She Z, et al. Insights into the epidemiology, pathogenesis, and therapeutics of nonalcoholic fatty liver diseases. Adv Sci (Weinh). 2018;6(4):1801585. 10.1002/advs.201801585 PMC6382298 30828530

[48] Méndez-Sánchez N, Bugianesi E, Gish RG (2022). Global multi-stakeholder endorsement of the MAFLD definition. Lancet Gastroenterol Hepatol.

