# Supplementary material for: Gender differences in the ideal cutoffs of visceral fat area for predicting MAFLD in China
Source: Lipids Health Dis. 2022 Dec 31;21:148. doi: 10.1186/s12944-022-01763-2 (PMC9805250; doi:10.1186/s12944-022-01763-2)
Supplement: Supplementary file 1 — Additional file 1: Supplementary Table 1. Comparison of baseline clinical data for patients in the training set and validation set. [file 12944_2022_1763_MOESM1_ESM.docx]

Supplementary Table 1. Comparison of baseline clinical data for patients in the training set and validation set

| **Variable** | **Training set**  **（n=3738）** | **Validating set**  **（n=1602）** | ***P*-value** |
| --- | --- | --- | --- |
| **Gender** |  |  | 0.182 |
| women | 1663 (44.49%) | 681 (42.51%) |  |
| men | 2075 (55.51%) | 921 (57.49%) |  |
| **Age, mean, (SD), year** | 53.24 ± 9.84 | 53.25 ± 9.50 | 0.974 |
| **BMI, mean, (SD), kg/m^2^** | 24.11 ± 3.03 | 24.18 ± 2.95 | 0.446 |
| **SBP, mean (SD), mmHg** | 125.86 ± 18.13 | 125.54 ± 17.50 | 0.550 |
| **DBP, mean (SD), mmHg** | 75.31 ± 11.30 | 75.25 ± 11.01 | 0.872 |
| **WC, mean, (SD), CM** | 81.91 ± 9.44 | 82.33 ± 9.56 | 0.140 |
| **WHR, mean, (SD)** | **0.87 ± 0.07** | **0.87 ± 0.07** | **0.268** |
| **WHtR, mean, (SD)** | **0.50 ± 0.05** | **0.51 ± 0.05** | **0.158** |
| **GGT, mean, (SD),, U/L** | 32.63 ± 32.50 | 33.48 ± 38.93 | 0.424 |
| **ALT, mean, (SD), U/L** | 23.19 ± 15.41 | 23.79 ± 15.45 | 0.200 |
| **AST, mean (SD), U/L** | 22.23 ± 9.22 | 22.46 ± 8.65 | 0.397 |
| **5-NT, mean, (SD), U/L** | 4.09 ± 2.05 | 4.19 ± 2.37 | 0.206 |
| **ALB, mean (SD), U/L, g/L** | 44.37 ± 7.97 | 44.46 ± 8.12 | 0.703 |
| **UA, mean (SD), mg/dL** | 361.95 ± 95.17 | 365.97 ± 93.52 | 0.161 |
| **BUN, mean (SD), umol/L** | 5.64 ± 1.39 | 5.59 ± 1.34 | 0.215 |
| **SCr, mean (SD), umol/L** | 66.76 ± 17.76 | 66.83 ± 16.73 | 0.891 |
| **TG, mean, (SD), mmol/L** | 1.89 ± 1.85 | 1.82 ± 1.41 | 0.177 |
| **TC, mean (SD), mmol/L** | 5.25 ± 1.03 | 5.27 ± 1.01 | 0.428 |
| **HDL-C, mean (SD), mmol/L** | 1.34 ± 0.33 | 1.32 ± 0.32 | 0.296 |
| **LDL-C, mean (SD), mmol/L** | 2.88 ± 0.76 | 2.92 ± 0.79 | 0.063 |
| **FPG, mean (SD), mmol/L** | 5.40 ± 1.46 | 5.49 ± 1.64 | 0.035 |
| **HbA1C, mean (SD), %** | 5.83 ± 1.00 | 5.86 ± 0.95 | 0.488 |
| **HB, mean (SD), g/L** | 144.81 ± 15.39 | 145.46 ± 15.49 | 0.164 |
| **PLT, mean (SD), 10^9^/L** | 229.62 ± 64.02 | 229.77 ± 63.85 | 0.941 |
| **WBC, mean (SD), 109/L** | 6.05 ± 1.60 | 6.08 ± 1.55 | 0.510 |
| **LFC, mean (SD), %** | 6.96 ± 5.20 | 7.18 ± 5.18 | 0.153 |
| **VFA, mean (SD), cm^2^** | 155.27 ± 78.34 | 156.96 ± 74.33 | 0.463 |
| **MAFLD** |  |  | 0.216 |
| No | 1952 (52.22%) | 807 (50.37%) |  |
| Yes | 1786 (47.78%) | 795 (49.63%) |  |
| **Virus hepatitis** |  |  | **0.809** |
| **No** | **3639 (97.3%)** | **1561 (97.4%)** |  |
| **Yes** | **99 (2.7%)** | **41 (2.6%)** |  |
| **Hypertension** |  |  | 0.480 |
| No | 3307 (88.47%) | 1428 (89.14%) |  |
| Yes | 431 (11.53%) | 174 (10.86%) |  |
| **Diabetes** |  |  | 0.706 |
| No | 3533 (94.52%) | 1510 (94.26%) |  |
| Yes | 205 (5.48%) | 92 (5.74%) |  |
| **Smoking** |  |  | 0.110 |
| No | 2835 (75.84%) | 1182 (73.78%) |  |
| Yes | 903 (24.16%) | 420 (26.22%) |  |
| **Drinking** |  |  | **0.526** |
| **No** | **2494 (66.72%)** | **1047 (65.36%)** |  |
| **light drinking** | **1142 (30.55%)** | **514 (32.08%)** |  |
| **Heavy drinking** | **102 (2.73%)** | **41 (2.56%)** |  |
| **Physical activity** |  |  | 0.152 |
| Low | 1239 (33.19%) | 530 (33.10%) |  |
| Moderate | 1281 (34.32%) | 588 (36.73%) |  |
| High | 1213 (32.49%) | 483 (30.17%) |  |

Data were presented as mean (SD) or n (%).

Data were presented as mean (SD) or n (%).

Continuous variables were represented as mean ± SD; categorical variables were expressed as numbers (percentages); the Kruskal-Wallis rank test was used for continuous variables and the chi-square test for categorical variables, and when the expected value was <10, the Fisher’s exact test was used.

Abbreviation: BMI, body mass index; WC, waist circumference; WHR, waist-to-hip ratio; WHtR, waist-to-Height ratio; SBP, systolic pressure; DBP, diastolic pressure; GGT, γ-glutamyl transpeptidase; ALT, alanine aminotransferase; AST, aspartate aminotransferase; 5-NT, 5'- nucleotidase; ALB, albumin; UA, uric acid; BUN, blood urea nitrogen; SCr, Serum creatinine; TG, triglyceride; TC, total cholesterol; HDL-C, high-density lipoprotein cholesterol; LDL-C, low-density lipoprotein cholesterol; FPG, fasting blood glucose; HbA1c, glycosylated hemoglobin; HGB, hemoglobin; PLT, platelet; WBC, white blood cells; LFC, Liver fat content; VFA, visceral fat area.
